# Clinical significance of *Nosocomiicoccus ampullae* isolated from blood cultures

**DOI:** 10.1128/spectrum.02179-23

**Published:** 2023-10-19

**Authors:** Victoria L. Campodónico, Tsigereda Tekle, Yehudit Bergman, Jennifer Lu, Emily Gisriel, Mark Romagnoli, Dimitrios Bourdas, Ann Hanlon, Carmila Manuel, Romney Humphries, Patricia J. Simner, Karen C. Carroll

**Affiliations:** 1 Division of Medical Microbiology, Department of Pathology, The Johns Hopkins University School of Medicine, Baltimore, Maryland, USA; 2 Department of Biomedical Engineering, Johns Hopkins University, Baltimore, Maryland, USA; 3 Center for Computational Biology, Whiting School of Engineering, Johns Hopkins University, Baltimore, Maryland, USA; 4 Department of Pathology, Microbiology and Immunology, Vanderbilt University Medical Center, Nashville, Tennessee, USA; 5 Division of Infectious Diseases, Department of Medicine, The Johns Hopkins University School of Medicine, Baltimore, Maryland, USA; University of Maryland School of Medicine, Baltimore, Maryland, USA

**Keywords:** *Nosocomiicoccus*, bloodstream infections, blood culture contaminant

## Abstract

*Nosocomiicoccus* species are infrequently isolated from clinical specimens and their clinical significance has not been evaluated. We retrospectively reviewed five cases in which *Nosocomiicoccus ampullae* was identified in a single blood culture set during routine culture. All patients were admitted for altered mental status and had several comorbidities. Patients improved without antibiotic therapy directed to *N. ampullae* or their condition declined due to underlying conditions, and *N. ampullae* isolation was not considered clinically significant in any case. Matrix-assisted laser desorption/ionization-time of flight mass spectrometry and/or 16S rRNA gene Sanger sequencing allowed genus identification, but whole genome sequencing was required for accurate species identification. All strains were susceptible to penicillin, oxacillin, cefoxitin, tetracycline, doxycycline, daptomycin, minocycline, clindamycin, nitrofurantoin, vancomycin, and linezolid; they showed high-level resistance to trimethoprim-sulfamethoxazole and erythromycin, and inducible resistance to clindamycin, applying Clinical and Laboratory Standards Institute *Staphylococcus* spp. guidelines with an extended incubation. They frequently carry genes associated with macrolide resistance. In conclusion, *N. ampullae* appears to have low pathogenicity and most probably represents a contaminant when isolated from a single blood culture set.

**IMPORTANCE:**

*Nosocomiicoccus* species are recently described as members of the *Staphylococcaceae* family. With their inclusion in commercial matrix-assisted laser desorption/ionization-time of flight mass spectrometry databases, *Nosocomiicoccus* species can now be identified when Gram-positive cocci in clusters are detected in positive blood cultures. However, their clinical significance is not known, making it difficult for the clinical microbiology laboratory to decide the extent of work-up. Based on our study, *Nosocomiicoccus* species demonstrate low pathogenicity and opportunistic potential. If isolated from a single blood culture set, limited work-up should be performed to an extent similar to other possible blood culture contaminants.

## OBSERVATION

*Nosocomiicoccus* species are Gram-positive, aerobic cocci, members of the *Staphylococcaceae* family (1). There are two species in this genus, *Nosocomiicoccus ampullae* (2) and *Nosocomiicoccus massiliensis* (3). A third species, “*Candidatus* Nosocomiicoccus stercorigallinarum”, identified as part of the chicken gut microbiome, has been proposed (4).

Reports on the isolation of these organisms are scarce and their clinical significance is uncertain (Table 1). *N. ampullae* was first described in 2008 after isolation from the surface of physiological saline bottles used repeatedly for topical washing of wounds (2). Later, it was isolated from a joint aspirate during routine diagnostic analysis (5), from a bloodstream infection in an adult patient (6), from the urine of an adult female with urinary symptoms (7), and from a Nigerian fermented soybean condiment (8). However, based on whole genome sequencing in a later study, it was proposed that the urinary strain was actually *N. massiliensis* (5). *N. massiliensis* was first isolated from the fecal microbiota of an individual living with HIV and experiencing acquired immunodeficiency syndrome in 2012 (3) and no additional reports have been published. In this study, we sought to determine the clinical significance of *N. ampullae* isolated from blood cultures. In addition, we evaluated their antimicrobial susceptibility profiles using several antimicrobial susceptibility testing (AST) methods.

**TABLE 1:** *Nosocomiicoccus* species isolated to date

Source	MALDI-TOF MS^*a*^	16S rRNA gene sequencing	16S rRNA PCR-DGGE^*a*^	WGS^*a*^	DNA G + C content (mol%)	References
Saline bottles	N/A^*a*^	*N. ampullae*	N/A	N/A	33.5	(2)
Urine	No ID	*N. ampullae*	N/A	*N. massiliensis*	36.2	(5, 7)
Joint fluid	No ID	N/A	N/A	*N. ampullae*	34.3	(5)
Blood	*N. ampullae*	N/A	N/A	N/A	N/A	(6)
Nigerian soybean	N/A	N/A	*N. ampullae*	N/A	N/A	(8)
Stool	No ID	N/A	N/A	*N. massiliensis*	36.4	(3)
Chicken stool				“*Candidatus* Nosocomiicoccus stercorigallinarum”	34.64	(4)

^*a*^ WGS: whole genome sequencing; PCR-DGGE: polymerase chain reaction-denaturing gradient gel electrophoresis; MALDI-TOF MS: matrix-assisted laser desorption/ionization-time of flight mass spectrometry; N/A: not available.

Cases in which *Nosocomiicoccus* spp. were isolated from blood cultures using the BACTEC system (BD Diagnostics, Inc., Sparks, MD, USA) and identified by matrix-assisted laser desorption/ionization-time of flight mass spectrometry (MALDI-TOF MS; Bruker Daltonics, Billerica, MA, USA) and/or 16S rRNA gene Sanger sequencing during standard clinical work-up at the Johns Hopkins Hospital Medical Microbiology Laboratory were found through database search. Medical records were reviewed for clinical presentation, microbiology results, antibiotic therapy, and clinical outcome.

Between April 2014 and May 2022, *Nosocomiicoccus* spp. were identified in blood cultures from five unique patients without central venous catheters. *Nosocomiicoccus* spp. were not identified from any other specimen sources. The clinical features of the patients in which *Nosocomiicoccus* spp. were isolated are summarized in Table 2. A sixth case of isolation of *Nosocomiicoccus* spp. from blood cultures was identified toward the end of the preparation of this manuscript. The patient characteristics are presented in Table 2, but no further description of this patient/isolate is provided.

**TABLE 2:** Clinical features of cases in which *Nosocomiicoccus ampullae* was isolated from blood

Case no.	Age/ sex	Presentation	Clinical diagnosis	Other comorbid conditions	Positive blood culture sets	Other cultured organisms in blood	MALDI ID (RUO)	16S Sanger sequencing ID	Antimicrobial therapy	Clinical outcome
1	88/F	Altered mental status	Dehydration, malnutrition	Hypertension, osteoarthritis, thyroidectomy, dementia	1/1, anaerobic bottle	None	N/A	*Nosocomiicoccus* sp. [*N. ampullae* (98.79%)]	None	Improved and discharged
2	71/M	Status epilepticus, fever, and vomiting	Hypoxic brain injury, bilateral hemispheric cortical ischemia	Diabetes mellitus hypertension, end stage renal disease on dialysis, peripheral vascular disease, severe depression, recent acute cholecystitis	1/1, aerobic bottle	None	No ID	*N. ampullae* (99.20%)	Piperacillin-tazobactam	Worsening neurological status. Terminally extubated, antibiotics and dialysis stopped. Discharged to hospice 4 d after admission
3	49/M	Altered mental status	Acute on chronic wound infection	Lymphedema	1/2, aerobic bottle	None	No ID	Unable to identify. Closely related to *Nosocomiicoccus* species [*N. massiliensis* (96.17%), *N. ampullae* (94.70%)]	Vancomycin, Unasyn	Acute symptoms resolved. Completed 9 d of Unasyn and discharged
4	77/M	Altered mental status, fever, abdominal pain	UTI: *Proteus mirabilis* and *Aerococcus urinae*, COVID-19	Obesity, benign prostatichyperplasia, hypertension, urinary and bowel incontinence, and dementia	1/2, aerobic bottle	None	*N. massiliensis* (scores: 1.72, 1.82)	Unable to identify. Closely related to *Nosocomiicoccus* species [*N. massiliensis* (95.99%), *N. ampullae* (94.88%)]	Vancomycin, cefepime, ceftriaxone	Improved mental status. Discharged 9 d after admission
5	67/M	Altered mental status	Hepatic encephalopathy, sepsis, hypernatremia, bilateral pulmonary embolism	Cirrhosis, hepatitis C, severe pulmonary hypertension, thrombocytopenia, left parietal meningioma, metastatic prostate adenocarcinoma	1/3, aerobic bottle	*Staphylococcus simulans* (3/3 sets)	*N. massiliensis* (scores: 1.71, 1.92)	Not sequenced	Vancomycin, cefepime	Worsening mental status, transitioning to comfort care. Death 8 d after admission
6^*a*^	64/M	Altered mental status	Hypothermia, hypernatremia AKI, rhabdomyolysis, dry gangrene due to frostbite	Alcoholism, vascular disease, dementia	1/2, aerobic bottle	None	No ID (score <1.7)	*N. ampullae* (99.40%)	Cefepime, metronidazole, vancomycin (for 1 d)	Improved mental status. Discharged 9 d after admission

^*a*^ Patient identified after WGS and antimicrobial susceptibility testing had been performed on the other isolates. Not included in isolate characterization.

Patients were 49–88 years of age (mean 69.3) and 80% were male. All patients were admitted for altered mental status and had various comorbid conditions. There was no overlap in time or hospital location between the patients when/where the blood cultures were drawn. Three blood isolates of *Nosocomiicoccus* spp. were from a single set out of two or three sets of blood cultures collected within 48 hours, and two from the only set collected. *Nosocomiicoccus* sp. was the only organism recovered in four cases and these cultures took between 43 and 59 hours to flag positive. Even though *Nosocomiicoccus* sp. has been previously described as aerobic or microaerophilic but unable to grow anaerobically (2), one of the isolates was recovered in the anaerobic bottle. One patient with *Nosocomiicoccus* sp. in one of three blood culture sets had *Staphylococcus simulans* in all three sets. Two patients were not diagnosed with infectious processes; one patient had signs of a skin infection without a definitive pathogen and one patient was diagnosed with severe acute respiratory syndrome coronavirus 2 infection and urinary tract infection caused by *Proteus mirabilis* and *Aerococcus urinae*.

All isolates were identified as Gram-positive cocci in clusters. Colonies showed a light-yellow pigment and no hemolysis; they were catalase and oxidase positive, consistent with previous reports of *N. ampullae* (2, 3). During standard of care work-up, MALDI-TOF MS was performed on four isolates; two were identified to the genus level (score <2.0) and two were unable to be identified. By 16S rRNA gene Sanger sequencing, one isolate was identified as *N. ampullae*, one as *Nosocomiicoccus* sp., two were unable to be identified, and one was not sequenced. Subsequently, all isolates underwent whole genome sequencing by Illumina MiSeq short-read sequencing (Illumina, San Diego, CA, USA) as previously described (9), and assembled using SPAdes (10). Nucmer (11) was then used to perform all pairwise alignments between each of the GenBank assemblies (nine sequences) and our isolate assemblies. Using the percent identities calculated by nucmer, the overall percent match between each pair was calculated. From the distance matrix of these nine sequences, a phylogenetic tree was generated, and all five isolates were determined to belong to *N. ampullae*.

AST was not performed or requested during the standard of care procedures but was performed retrospectively for this study. Since *Nosocomiicoccus* spp. were recently assigned to the *Staphylococcaceae* family (1) and we demonstrated that our isolates grew better under ambient air than under air supplemented with 5% CO_2_ (Fig. S1), AST was performed following the Clinical and Laboratory Standards Institute (CLSI) guidelines and interpretation for testing *Staphylococcus* spp. other than *S. aureus*, *S. lugdunensis*, *S. epidermidis*, *S. pseudointermedius*, and *S. schleiferi* (12). These interpretative criteria were chosen due to the phylogenetic relatedness between *Nosocomiicoccus* sp. and members of the *Staphylococcaceae* family, as it has been used for *Micrococcus* spp. (13); however, interpretative criteria have not been established for *Nosocomiicoccus* spp. AST using the BD Phoenix automated microbiology system (BD Diagnostic Systems, Sparks, MD, USA) was first attempted; however, the isolates failed to grow overnight in the system. Therefore, we performed AST by reference broth microdilution (BMD) in cation-adjusted Mueller-Hinton broth, and gradient diffusion (Etest; bioMerieux, Marcy-l’Étoile, France) and by disk diffusion (DD; BBL disks, BD Diagnostic Systems, Sparks, MD, USA) in Mueller-Hinton agar with reading performed following 48 hours of incubation at 35°C in ambient air, instead of after the 16–24 hours of incubation recommended by CLSI M100 (Table S1) (12, 14, 15). Based on results from the reference BMD and applying *Staphylococcus* spp. interpretations, *N. ampullae* isolates were 100% susceptible to penicillin, oxacillin, tetracycline, doxycycline, daptomycin, minocycline, clindamycin, vancomycin, and linezolid. Most strains (80%) were resistant to trimethoprim-sulfamethoxazole. By DD, all strains were susceptible to cefoxitin and nitrofurantoin, and four were susceptible to moxifloxacin. Results from BMD and DD were not 100% concordant for erythromycin. By BMD, three strains tested susceptible, one demonstrated an intermediate MIC, and one was resistant to erythromycin; by DD, there was one susceptible strain, one was intermediate, and three were resistant. Further studies are needed to determine which method is more suitable to determine erythromycin resistance within these strains. Four strains showed inducible clindamycin resistance by DD (D-test positive) (Table S1) (12). Clindamycin inducible resistance by BMD was not tested. Interestingly, antimicrobial resistance screening using ABRicate (16) on the isolate assemblies revealed four strains that showed resistance to erythromycin and were found to carry the *ermY* gene (Table S1). This gene encodes for erythromycin ribosome methylase proteins responsible for macrolide resistance (17) and it was also found in the published sequencing data from the *N. ampullae* strain isolated from joint fluid (5).

None of the patients in this study received directed antimicrobial therapy for *Nosocomiicoccus* spp. One patient improved with supportive care without receiving antimicrobial treatment, two patients improved while on antibiotics that, although potentially active against *N. ampullae*, were directed toward other infections*,* and two patients developed worsening mental status due to underlying conditions with one discharged to hospice care and one expiring on day 8 of admission. Although it is difficult to completely rule out the pathogenic role of the isolates recovered from these patients, no providers noted *Nosocomiicoccus* spp. to be clinically significant and all patients had other conditions that could explain their symptoms.

In conclusion, *N. ampullae* appears to have low pathogenicity and opportunistic potential and most probably represents a contaminant when isolated from a single blood culture set. MALDI-TOF MS and sequencing of the first ~500 base pairs of the 16S rRNA gene may not allow discrimination between different species within the *Nosocomiicoccus* genus. If species-level identification is desired, the oxidase test could differentiate between *N. massiliensis* (negative) and *N. ampullae* (positive). Additionally, *N. ampullae* demonstrates high resistance rates to trimethoprim-sulfamethoxazole, erythromycin and inducible resistance to clindamycin, and they can possess antimicrobial resistance genes associated with macrolide resistance.

## Data Availability

Whole genome sequences of the *Nosocomiicoccus ampullae* isolates used in this study can be found at accession: PRJNA980722, ID: 980722.

## SUPPLEMENTAL MATERIAL

Supplemental figure legend spectrum.02179-23-s0001.docx Legend for Fig. S1.

Fig. S1 spectrum.02179-23-s0002.tif Growth of *Nosocomiicoccus ampullae* isolates on blood agar.

Table S1 spectrum.02179-23-s0003.docx Antimicrobial susceptibility testing of *Nosocomiicoccus ampullae*.

ASM does not own the copyrights to Supplemental Material that may be linked to, or accessed through, an article. The authors have granted ASM a non-exclusive, world-wide license to publish the Supplemental Material files. Please contact the corresponding author directly for reuse.
