# Supplementary material for: Clinical significance of Nosocomiicoccus ampullae isolated from blood cultures
Source: Microbiol Spectr. 2023 Oct 19;11(6):e02179-23. doi: 10.1128/spectrum.02179-23 (PMC10715106; doi:10.1128/spectrum.02179-23)
Supplement: Supplemental figure legend — Legend for Fig. S1. [file spectrum.02179-23-s0001.docx]

**Figure S1. Growth of *Nosocomiicoccus ampullae* isolates on blood agar**

**Figure S1 legend.** Cultures of *Nosocomiicoccus ampullae* isolates showing enhanced growth, particularly for isolates #1 and #2, in ambient air compared to air supplemented with 5% CO_2_ after 24 hours of incubation.
