# Supplementary figures and images for: Clinical significance of Nosocomiicoccus ampullae isolated from blood cultures
Source: Microbiol Spectr. 2023 Oct 19;11(6):e02179-23. doi: 10.1128/spectrum.02179-23 (PMC10715106; doi:10.1128/spectrum.02179-23)

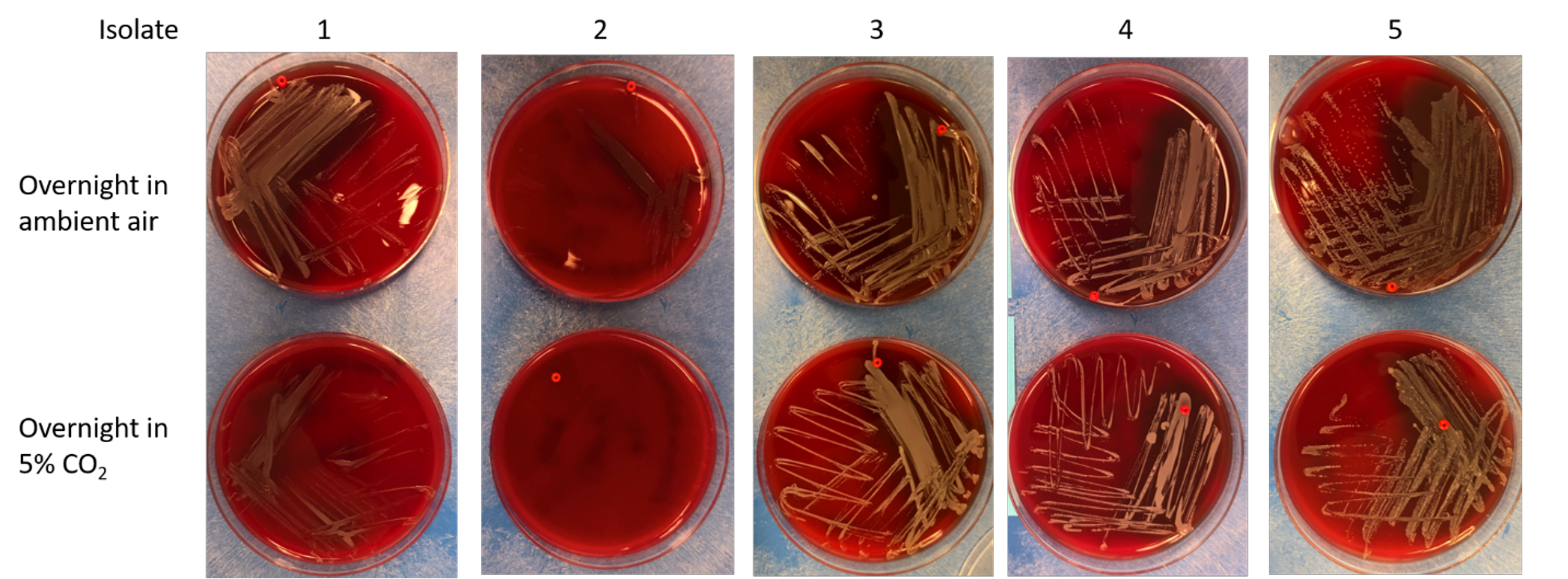

Supplement: Fig. S1 — Growth of Nosocomiicoccus ampullae isolates on blood agar. [file spectrum.02179-23-s0002.tif]
