# Supplementary material for: Clinical significance of Nosocomiicoccus ampullae isolated from blood cultures
Source: Microbiol Spectr. 2023 Oct 19;11(6):e02179-23. doi: 10.1128/spectrum.02179-23 (PMC10715106; doi:10.1128/spectrum.02179-23)
Supplement: Table S1 — Antimicrobial susceptibility testing of Nosocomiicoccus ampullae. [file spectrum.02179-23-s0003.docx]

| **Table S1. Antimicrobial susceptibility testing of *Nosocomiicoccus ampullae* by broth microdilution​ (BMD), Etest and disk diffusion following CLSI guidelines and interpretations for *Staphylococcus* spp. with an extended incubation of up to 48 hours.** | | | | | | | | | | | | | | | | | |
| --- | --- | --- | --- | --- | --- | --- | --- | --- | --- | --- | --- | --- | --- | --- | --- | --- | --- |
| **Isolate** | **AST method** | **CPT** | **LIN** | **VAN** | **ERY** | **PEN** | **CLN** | **DAP** | **TET** | **MIN** | **SXT** | **OXA** | **CFX** | **DOX** | **FUR** | **MOX** | ***ermY* gene** |
| **1** | **BMD** | 4 | < 0.25 | < 0.5 | < 0.5 | < 0.015 | ≤ 0.12^#^ | ≤ 0.12 | ≤ 1 | ≤ 1 | ≤ 0.5 | ≤ 0.12 | ≤ 0.25 | ≤ 1 |  |  | + |
|  | **Etest** |  |  | 0.5 | 0.25 |  | ≤ 0.25 |  | 0.25 |  | 0.125 | 1 |  |  |  | 0.5 |  |
|  | **DD** |  |  |  | R |  | R* |  | S |  | I |  | S |  | S | S |  |
| **2** | **BMD** | ≤ 0.5​ | 0.5​ | ≤ 0.5​ | > 8​ | ≤ 0.015 | ≤ 0.12^#^​ | ≤ 0.12​ | ≤ 1​ | ≤ 1​ | > 8​ | ≤ 0.12​ | ≤ 0.25 | 2 |  |  | + |
|  | **Etest** |  |  | 1 | > 256 |  | 1 |  | 0.25 |  | 8 | 0.5 |  |  |  | 0.25 |  |
|  | **DD** |  |  |  | R |  | R* |  | S |  | R |  | S |  | S | S |  |
| **3** | **BMD** | ≤ 0.5 | 1 | ≤ 0.5 | ≤ 0.5 | 0.03 | ≤ 0.12​^#^ | ≤ 0.12​ | ≤ 1​ | ≤ 1​ | > 8 | ≤ 0.12​ | 0.5 | ≤ 1​ |  |  | - |
|  | **Etest** |  |  | 1 | 0.25 |  | 0.25 |  | 0.5 |  | >32 | 0.125 |  |  |  | > 32 |  |
|  | **DD** |  |  |  | S |  | S* |  | S |  | R |  | S |  | S | R |  |
| **4** | **BMD** | ≤ 0.5 | 0.5 | ≤ 0.5 | ≤ 0.5 | 0.03 | ≤ 0.12​^#^ | ≤ 0.12​ | ≤ 1​ | ≤ 1​ | > 8 | ≤ 0.12​ | 0.5 | ≤ 1​ |  |  | + |
|  | **Etest** |  |  | 1 | > 256 |  | 1 |  | 0.25 |  | > 32 | 0.25 |  |  |  | 0.25 |  |
|  | **DD** |  |  |  | R |  | R* |  | S |  | R |  | S |  | S | S |  |
| **5** | **BMD** | ≤ 0.5 | 1 | ≤ 0.5 | 1 | ≤ 0.015 | ≤ 0.12^#^​ | ≤ 0.12​ | ≤ 1​ | ≤ 1​ | > 8 | 0.12​ | < 0.25 | ≤ 1​ |  |  | + |
|  | **Etest** |  |  | 0.5 | 1 |  | 0.5 |  | 0.25 |  | > 32 | 0.5 |  |  |  | 0.5 |  |
|  | **DD** |  |  |  | I |  | R* |  | S |  | R |  | S |  | S | S |  |

BMD: broth microdilution; DD: disk diffusion; CPT: ceftaroline, LIN: linezolide, VAN: vancomycin, ERY: erythromycin, PEN: penicillin, CLN: clindamycin, DAP: daptomycin, TET: tetracycline, MIN: minocycline, SXT: trimethoprim-sulfamethoxazole, OXA: oxacillin, CFX: cefoxitin, DOX: doxycycline, FUR: nitrofurantoin, MOX: moxifloxacin

S: susceptible, I: intermediate resistance, R: resistant

^#^ Inducible clindamycin resistance was not tested by BMD

*Only inducible clindamycin resistance was tested by disk diffusion.
